# Access to T-Cell Redirecting Therapies in Multiple Myeloma: Patient, Caregiver, and Physician Perspectives on Awareness and Barriers

**DOI:** 10.3390/cancers18152412

**Published:** 2026-07-27

**Authors:** Zalak Shah, Myra Robinson, Abena Prempeh, Nicole Serapin, Ami Ndiaye, Peter M. Voorhees, Manisha Bhutani

**Affiliations:** 1 Atrium Health Levine Cancer Institute, Atrium Health Wake Forest Baptist Comprehensive Cancer Center, Charlotte, NC 27157, USA; 2 Department of Biostatistics and Data Sciences, Atrium Health Levine Cancer Institute, Atrium Health Wake Forest Baptist Comprehensive Cancer Center, Charlotte, NC 27157, USA; 3 Meharry Medical College, Nashville, TN 37208, USA; 4 Department of Cancer Medicine, Atrium Health Levine Cancer Institute, Wake Forest University School of Medicine, Charlotte, NC 28204, USA

**Keywords:** multiple myeloma, t-cell redirecting therapies, access to care, treatment barriers, patient perspectives, caregiver perspectives, physician perspectives, healthcare disparities

## Abstract

**Background**: T-cell redirecting therapies (TCRTs), including CAR T-cell therapies and bispecific antibodies, have transformed treatment for relapsed/refractory multiple myeloma (MM), yet real-world adoption remains limited by awareness and access barriers across patients, caregivers, and physicians. **Methods**: We conducted a cross-sectional study (June–September 2025) using IRB-approved REDCap surveys distributed to patients with MM (via MyChart), caregivers of TCRT recipients (via email), and community physicians (via email). Surveys assessed TCRT familiarity, perceived barriers, and access challenges. The primary objective was to compare TCRT utilization across racial groups among previously treated patients. **Results**: A total of 428 respondents participated (346 patients, 51 caregivers, 31 physicians). Among patients, 18% were Black. Overall, 26% were unfamiliar with TCRT, with higher unawareness among Black patients (32% vs. 25%) and those with ≤high school education (42% vs. 23%). Higher education was associated with twice the odds of TCRT utilization (*p* = 0.05). Among patients reporting prior MM treatment, TCRT utilization was similar across racial groups (39% Black vs. 38% non-Black). Among patients reporting TCRT receipt, Black patients more frequently reported receiving therapy through clinical trials compared with non-Black patients (44% vs. 34%). Among caregivers, 53% reported effects on mental/emotional wellness, 61% moderate-to-high stress during treatment, and 33% provided >40 h/week of care during month one. Among physicians, 71% referred patients for TCRT, but 48% reported <25% of referrals resulted in treatment. Patient hesitancy was the most cited referral barrier. **Conclusions**: Awareness gaps disproportionately affect Black patients and those with lower education. Caregivers experience substantial emotional and time burden. Patient hesitancy remains the primary barrier to TCRT referrals. Clinical trials may serve as an alternative access pathway for some patients. Multi-level interventions targeting education, caregiver support, and care coordination are needed to improve equitable TCRT access.

## Simple Summary

Newer treatments that use the body’s immune system to fight multiple myeloma have improved outcomes for people with myeloma that have come back or stopped responding to other treatments. Despite their availability, many patients face challenges in learning about and receiving these therapies. This study surveyed patients, caregivers, and community physicians to better understand awareness, access, and barriers to treatment. The study found that Black patients and those with lower levels of education were less familiar with these therapies, although treatment use was similar across racial groups. Caregivers reported significant emotional stress and time demands, and physicians identified patient hesitancy as the most common reason that limited referrals to institutions that provide these therapies. These findings highlight the need for better patient education, stronger caregiver support, and improved coordination of care to help ensure equitable access to these efficacious treatments.

## 1. Introduction

T-cell redirecting therapies (TCRTs), including BCMA-directed CAR T-cell therapies and bispecific antibodies, have led to unprecedented response rates in heavily pretreated patients with multiple myeloma who previously had limited options, though resistance mechanisms remain an evolving challenge [1,2]. Despite this efficacy, real-world access remains limited due to requirements for certified tertiary centers, manufacturing delays for CAR T products, step-up dosing protocols for bispecific antibodies, and high costs [3,4]. Geographic, socioeconomic, and racial disparities further limit access. Patients in rural regions often live far from treatment centers, and lower-income patients represent only 7.3% of CAR T recipients [5]. African Americans comprise just 6–18% of participants in pivotal TCRT trials for multiple myeloma, and only 35.9% reside in counties with access to TCRT trials [6,7]. Among patients who receive CAR T-cell therapy, real-world studies have shown comparable outcomes across racial groups [8].

TCRTs carry risks of cytokine release syndrome and neurological toxicities, necessitating reliable caregiver support and management at specialized centers [9]. Caregivers often face substantial distress due to prognostic uncertainty, temporary relocation and lodging demands near treatment centers, and the need to recognize and manage unfamiliar and sometimes severe toxicities [10,11]. Despite their critical role, caregivers’ psychosocial experiences across the treatment trajectory remain largely uncharacterized in the literature [12,13]. Community physicians, although not always specialists, play an essential role in identifying patients and facilitating referrals for TCRT. Referring physicians most often cite financial toxicity as the primary barrier, whereas treating oncologists emphasize logistical concerns such as leukapheresis slot availability and hospitalization requirements [14].

Existing disparities in treatment access and outcomes illustrate the importance of understanding how awareness influences both access to TCRT and treatment decision-making. While barriers to TCRT access have been identified at system and policy levels, there is limited understanding of awareness, perceptions, and practical challenges from the perspectives of patients, caregivers, and community physicians simultaneously.

This study aimed to assess awareness, perceived barriers, and practical challenges surrounding TCRT among three key stakeholder groups: patients with multiple myeloma, their caregivers, and community-based physicians. We sought to identify disparities in awareness and utilization across racial and educational groups, quantify caregiver burden and distress, and characterize physician-reported barriers to referral and post-treatment management. By examining these perspectives simultaneously, we aimed to identify actionable targets for interventions to enhance TCRT education, improve care coordination, and strengthen support systems across all stakeholders.

## 2. Methods

### 2.1. Study Design and Setting

This prospective, cross-sectional survey study was conducted at Atrium Health Wake Forest Baptist Comprehensive Cancer Center (AHWFBCCC). The study and all associated materials were approved by the Institutional Review Board at Wake Forest University School of Medicine with a waiver of informed consent and waiver of HIPAA authorization, as participant lists were generated from data collected during standard clinical care and all survey responses were anonymous and non-identifiable.

### 2.2. Survey Development and Content Validity

Three separate survey instruments were developed internally. Survey content was informed by a literature review of the existing literature on barriers to advanced cancer therapies and expert consensus regarding key domains of awareness, access, and decision-making for TCRT. The surveys underwent content validity review by clinical experts in multiple myeloma and TCRT to ensure relevance and comprehensiveness. Formal psychometric validation, including cognitive interviews, test–retest reliability assessment, or evaluation of internal consistency, was not performed because these surveys were designed to explore stakeholder experiences, perceptions, and barriers related to TCRT rather than to function as standardized measurement tools.

The patient survey consisted of 25 items covering: demographic characteristics, multiple myeloma treatment history, familiarity with TCRT, perceived risks and benefits, clinical trial participation attitudes, access barriers, and informational needs. The caregiver survey included 22 items assessing: relationship to patient, caregiving responsibilities and time commitment, emotional and logistical burden, knowledge of TCRT, challenges in accessing treatment centers, and support needs. The physician survey contained 21 items evaluating: practice characteristics, patient population demographics, familiarity with TCRT, referral practices and barriers, post-TCRT management comfort, and recommendations for improving access. Complete survey instruments are provided in the Supplementary Materials (Figures S3–S5).

### 2.3. Participant Identification and Survey Distribution

The survey was distributed to three participant groups: (1) patients aged 18 years or older with self-reported multiple myeloma, (2) self-reported designated caregivers of patients with multiple myeloma who had received or were receiving TCRT, and (3) community-based physicians who treat MM patients and whose practices did not offer CAR T-cell therapy or step-up dosing of bispecific antibodies. All participants were required to be able to complete the survey in English. There were no exclusion criteria.

Eligible patients were identified through a structured query of the electronic health record within the Atrium Health Wake Forest Baptist system. Patients received a secure survey link via MyChart, the institution’s patient portal, accompanied by a message explaining the study’s purpose and encouraging them to share the survey link with their caregivers.

Caregivers were identified from two sources: (1) the Transplant and Cellular Therapy program’s list of designated caregivers for patients receiving TCRT, and (2) patient-initiated sharing of the survey link via MyChart. Identified caregivers received the survey link via email.

Clinicians practicing in community settings within the Advocate Health system were identified through institutional referral networks. Physicians received the survey link via email with a description of the study purpose and eligibility criteria.

The surveys were distributed in June 2025, and responses were collected through September 2025 using REDCap (Research Electronic Data Capture), a secure web-based platform for managing online surveys and databases. The survey landing page outlined the purpose, risks, and benefits of participation, and completion of the survey constituted implicit informed consent. Participation was voluntary; no compensation was offered, and participants could skip questions or withdraw at any time without consequence. All data, including demographics and TCRT exposure, were self-reported through the surveys. No identifiable information was collected, and no email addresses or other identifiers were linked to survey responses. All survey data were stored in Enterprise REDCap and accessed only on secure Atrium Health devices. Data were viewed and analyzed exclusively by authorized study personnel, and no protected health information was included in the survey dataset, ensuring complete participant anonymity.

### 2.4. Study Variables and Definitions

The primary outcome was patient-reported utilization of TCRT among respondents who reported having received prior therapy for multiple myeloma, stratified by race (Black vs. non-Black). Prior multiple myeloma treatment, as self-reported by patients, was used as a proxy for TCRT eligibility, as current FDA approval criteria for BCMA-directed therapies generally require at least one prior line of therapy.

Key independent variables included race (self-reported as Black, White, Asian, or other) and educational attainment (dichotomized as high school or less vs. some college or higher). For caregivers, key outcome variables included caregiving time commitment (hours per week), emotional stress level (low, moderate, high), specific challenges faced (emotional distress, isolation, appointment coordination, financial burden), and barriers to locating treatment centers. For physicians, key outcome variables included proportion of patients from marginalized communities, number of patients referred for TCRT, proportion of referred patients who received TCRT, comfort level with post-TCRT management, and perceived barriers to access (geographic, financial, logistical).

### 2.5. Statistical Analysis

Descriptive statistics were used to characterize respondent demographics and survey responses. Categorical variables were summarized using frequencies and percentages. Comparisons between racial groups (Black vs. non-Black) and educational groups (high school or less vs. higher education) were performed using Fisher’s exact tests for categorical variables. Statistical significance was defined as *p* < 0.05 and *p*-values were used to identify potential associations warranting further investigation. Logistic regression was used to evaluate associations between race or education with patient-reported TCRT utilization. In addition to the key covariates of interest (race, education), factors evaluated in model selection included patient reported age (<60 vs. ≥60 years), sex, residence area (urban/suburban vs. rural), and insurance status (commercial/private vs. other). The non-key factors were evaluated in individual models, with planned backward elimination (elimination criteria *p* ≥ 0.05) and forward selection (entry criteria *p* < 0.05) to identify the base model for the outcome. The key covariates of interest (race, education) were then both added to the base model that was determined with multivariable model selection.

All analyses were conducted using SAS v9.4. Missing data were not imputed; analyses were restricted to participants with complete data for the variables of interest. Patterns of missingness were summarized by group with frequencies and proportions.

No formal sample size calculation was performed given the exploratory, descriptive nature of this study. The target was to recruit all eligible patients with multiple myeloma receiving care at AHWFBCCC during the study period, all identifiable caregivers of TCRT recipients, and a convenience sample of community physicians in the referral network.

## 3. Results

### 3.1. Participant Characteristics

Between 1 June and 30 September 2025, 428 respondents initiated the surveys across three stakeholder groups: 346 patients with multiple myeloma, 51 caregivers of patients receiving TCRT, and 31 community-based physicians. The survey was distributed to 1770 patients in June 2025 via MyChart (20% response rate). Approximately 65 community physicians and 150 caregivers were invited to participate, with 31 physicians and 51 caregivers completing the survey (48% and 34% response rate, respectively). Among the patient respondents, 18% identified as Black, 79% White, 1% Asian, 1% other race, and 1% preferred not to answer. The majority of patients were ≥60 years old (79%), and 56% of patients were male. Most patients (82%) had an education level greater than high school, and 68% reported living in an urban or suburban area (Table 1). There was an underrepresentation of Black patients among respondents (18%) com-pared to the target study population (34%) (Table S1).

Of the caregiver respondents, 12% identified as Black and the majority (63%) were 65 years or older. Most (75%) caregivers were female, reported that they were spouses or partners to the patient (82%), and had greater than high school level education (88%, Table 2).

Of the physician respondents, specialties included hematology/medical oncology (39%), medical oncology (29%), hematology (6%), internal medicine (3%), or other (23%). The experience levels from respondents varied, with 29% having 10 years or less practicing, 35% with 11–20 years, and 35% with more than 20 years practicing medicine.

### 3.2. Patient Survey Responses

**Figure 1:**
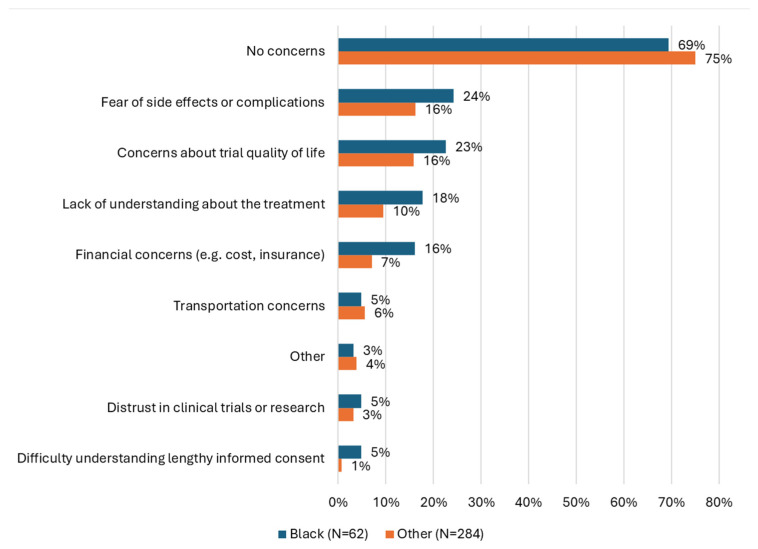
Patient-Reported Concerns About Participation in Clinical Trials by Race. Horizontal bar chart comparing reported concerns related to participation in clinical trials among Black (N = 62) and non-Black (N = 284) patient respondents. Black respondents more frequently reported concerns related to side effects, quality of life, understanding of treatment, and financial burden. Multiple responses were permitted.

More than a quarter of patient-respondents (26%, 90 of 346) endorsed being unfamiliar with TCRT. Lack of awareness was higher among Black patients (32%) compared to non-Black patients (25%), and among those with high school education or less (42%) versus those with higher education (23%). Nearly three-quarters of respondents (74%) had no concern with participating in clinical trials, with similar rates across racial groups (69% vs. 75% in Black vs. non-Black respondents). Reported clinical trial participation concerns by race are shown in Figure 1, highlighting higher frequencies of specific concerns among Black respondents (Table S2).

**Figure 2:**
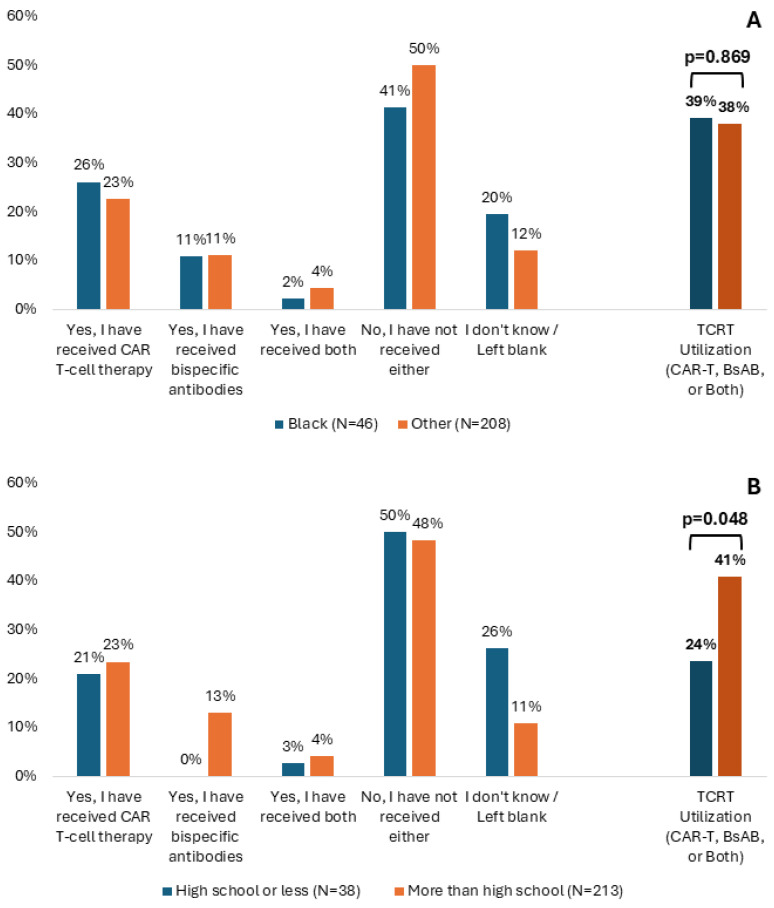
Patient-Reported Utilization of T-Cell Redirecting Therapy (TCRT) by Race and Education. (**A**) Utilization by race among patients with prior multiple myeloma treatments. (**B**) Utilization by education level. Percentages represent respondents in each category. Abbreviations: BsAb—bispecific antibody.

Among respondents who reported prior treatment for MM (n = 254, 73.4%), TCRT utilization was similar between Black and non-Black patients (39% vs. 38%, respectively; *p* = 0.87). Black patients reported similar TCRT utilization patterns to those of other races, with 28% receiving CAR T cell therapy and 13% bispecific antibodies, compared with 27% and 15%, respectively, among patients of other races (Figure 2A). With logistic regression, there was no significant association between race and TCRT utilization [unadjusted OR (Black vs. non-Black) 1.1, 95% CI: 0.5–2.0, *p* = 0.88].

Patients with higher educational attainment reported higher TCRT utilization patterns to those with lower educational attainment, with 28% receiving CAR T cell therapy and 17% bispecific antibodies, compared with 24% and 3%, respectively, among patients with high school or less (Figure 2B). Higher educational attainment was associated with greater odds of TCRT utilization, with participants reporting education beyond high school having approximately twice the odds of receiving TCRT compared to those with a high school education or less (unadjusted OR 2.2, 95% CI: 1.0–4.9, *p* = 0.049).

Although age, sex, area, and insurance were also evaluated for multivariable model selection to determine adjusted models, no associations were identified in individual models (Table S3). Therefore, the final logistic regression model included just race and education. There was no significant association between race and TCRT utilization, even after adjusting for education level (adjusted OR 1.1, 95% CI: 0.6–2.1, *p* = 0.797), but the association between education and TCRT utilization remained significant (adjusted OR 2.2, 95% CI: 1.0–5.0, *p* = 0.048).

Among respondents who reported receiving TCRT (n = 97), a greater proportion of Black patients reported receiving TCRT as part of a clinical trial compared with non-Black respondents (44% vs. 34%). Among patients who reported receiving TCRT, 88% reported no challenges accessing therapy. Similar proportions of this finding were seen across racial groups (Black: 89%, non-Black: 87%). Additionally, most respondents (82%) stated they would recommend TCRT without reservation, again with comparable rates between Black and non-Black patients (78% vs. 84%, respectively).

Patterns of missingness for key patient-reported outcomes are included in the Supplementary Materials (Tables S4 and S5). Notably, higher proportions of Black (20%) and less educated respondents (26%) answered “I don’t know” or left the utilization of TCRT question blank compared to non-Black (12%) and more educated respondents (11%), respectively.

### 3.3. Caregiver Survey Responses

**Figure 3:**
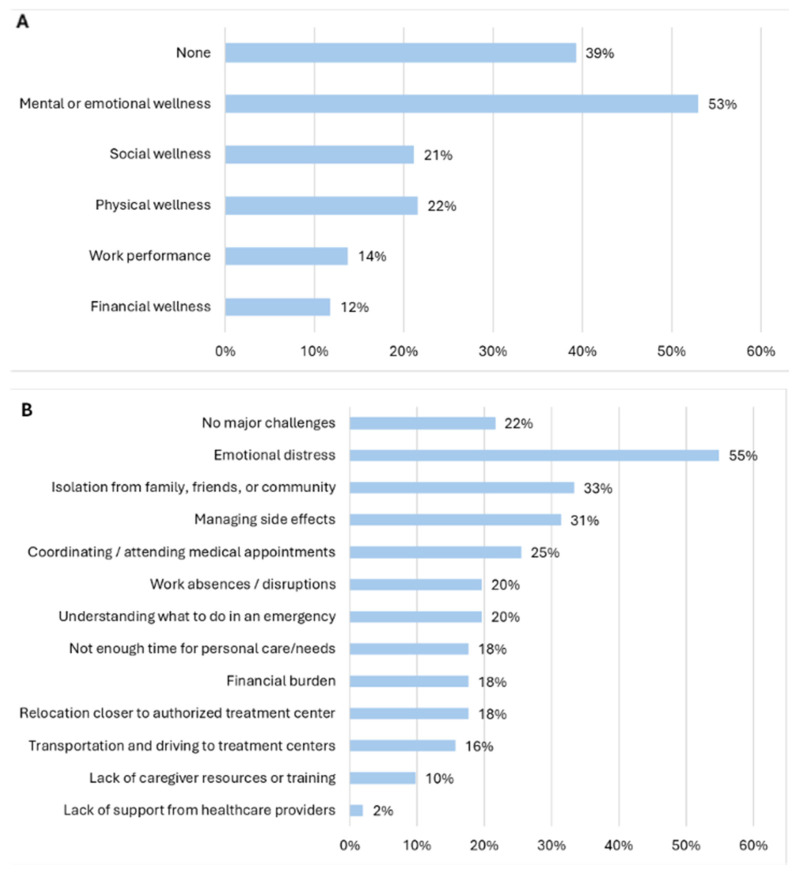
Caregiver-Reported Wellness Impact and Challenges During T-Cell Redirecting Therapy (TCRT). (**A**) Impact of caregiving on wellness domains, with mental or emotional well-being most affected. (**B**) Caregiver-reported challenges during TCRT, most frequently including emotional distress, social isolation, and managing treatment-related side effects. Percentages represent respondents selecting each item; multiple responses were permitted.

Among caregivers, 22% reported no prior knowledge of TCRT. The vast majority (90%) reported no difficulty in identifying a TCRT treatment center. Over half of caregivers (53%) reported that their mental or emotional wellness was affected by their caregiving role (Figure 3A), and 61% experienced moderate to high emotional stress during their care recipient’s TCRT treatment (Figure S1). One-third of caregivers (33%) reported spending more than 40 h per week on caregiving responsibilities during the first month of TCRT.

The most frequently cited challenge was emotional distress (55%), followed by isolation from family, friends, and community (33%) and managing treatment-related side effects (31%, Figure 3B).

### 3.4. Physician Survey Responses

**Figure 4:**
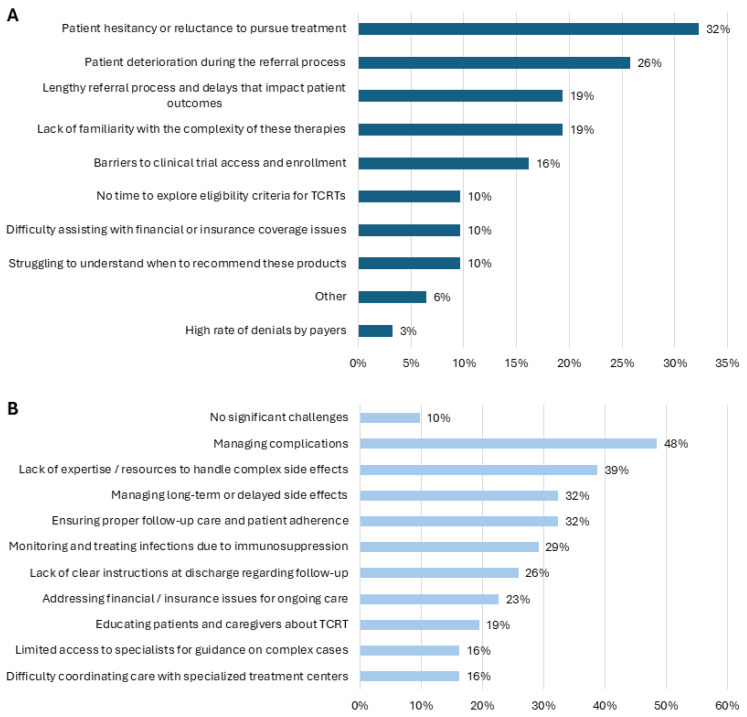
Physician-Reported Barriers to Referral and Post-TCRT Care Challenges. (**A**) Reported barriers when considering referral for T-cell redirecting therapy (TCRT), most commonly including patient hesitancy and clinical deterioration during the referral process. (**B**) Challenges faced in the care of patients after TCRT, most frequently including management of complications, limited expertise for complex side effects, and long-term toxicity management. Percentages represent respondents selecting each item; multiple responses were permitted.

Physician respondents reported wide variation in MM populations with 16% treating fewer than 10% of patients and 23% treating more than 50% of patients from marginalized communities. Referral to TCRT was common (71%), with all hematologists reporting referrals, compared to 86% referred among medical oncologists and 57% referred among non-oncology specialties; however, nearly half (48%) reported that fewer than a quarter of referred patients proceeded with treatment. Patient hesitancy or reluctance to pursue treatment was the most frequently cited barrier physicians encountered when considering TCRT referral (Figure 4A). When asked about the role of location in TCRT access, 74% of physicians indicated that geography plays a moderate or major role, and improved transportation and logistical support for patients was the most frequently selected recommendation (58%) for improving TCRT access in communities.

Although 65% had provided care to patients after TCRT, nearly one-third (29%) expressed low confidence (“not very comfortable” or “not comfortable at all”) in managing post-TCRT care. The most frequent post-TCRT challenge was managing complications (48%; Figure 4B). Physician survey responses regarding recommendations for improving access to TCRT in their communities are summarized in Figure S2.

## 4. Discussion

This multi-stakeholder survey study reveals interconnected barriers to access and awareness of TCRT among patients with multiple myeloma, their caregivers, and community physicians. Over one-quarter of patients were unfamiliar with TCRT, with significantly higher rates among Black patients and those with lower education. Educational attainment was the only patient-reported factor significantly associated with TCRT utilization, with higher education conferring twice the odds of receiving therapy. This association likely reflects multiple mechanisms. Patients with higher education may have greater health literacy, facilitating engagement with complex treatment information, as well as higher rates of MyChart adoption, which may have influenced survey participation [15,16]. MyChart adoption was not directly measured, so this possibility remains speculative. Patients with higher education may better recall exposure to TCRT, including recognition of therapy names or treatment categories, introducing potential recall bias [17]. Notably, existing CAR T-cell educational materials are typically written at 8th to 12th grade reading levels, well above the 6th grade level recommended by the American Medical Association [18]. Strategies such as incorporating plain-language summaries and visual aids may improve comprehension and support informed decision-making among diverse patient populations [19]. Development of these materials using input from patients and caregivers may help ensure that educational resources address the information gaps and concerns of individuals considering TCRT [20].

Although lack of awareness was higher among Black patients, TCRT utilization did not differ across racial groups, suggesting that physician-driven referral practices and clinical trial availability at our center may help mitigate gaps in patient-initiated engagement. This is further supported by the finding that Black TCRT recipients more frequently accessed therapy through clinical trials, indicating that trials may serve as an alternative access pathway when standard-of-care TCRT faces greater barriers [7]. However, in settings without robust clinical trial infrastructure or proactive referral practices, these awareness gaps could translate into differences in utilization. Improving equitable access to TCRT will require education-focused interventions, such as revising materials to appropriate reading levels, alongside consistent referral pathways and equitable trial access across diverse practice settings.

Our data on caregiver burden adds important quantitative evidence to a literature that has been largely qualitative. More than half of caregivers reported mental or emotional impacts. Nearly two-thirds experienced moderate-to-high stress, and one-third provided over 40 h of care per week, equivalent to a full-time job. These findings align with emerging CAR T-cell caregiver studies demonstrating persistent depressive symptoms and quality-of-life impairments at 180 days post-treatment [10,12,21]. The intensity of caregiving demands mirrors the broader oncology literature, where caregivers providing higher levels of support are more likely to report negative outcomes and postpone their own healthcare needs [22]. Emotional distress and isolation from family and community were the most frequently cited challenges, consistent with qualitative studies describing caregivers as ‘invisible sufferers’ facing long-term psychological distress [13].

These demands are amplified by TCRT-specific requirements, including prolonged proximity to treatment centers, around-the-clock caregiver availability, and learning to recognize unique toxicities such as cytokine release syndrome and neurotoxicity. The shift toward outpatient TCRT administration, while reducing hospitalization costs and improving patient autonomy, may paradoxically intensify caregiver burden by transferring monitoring and toxicity management responsibilities from inpatient nursing staff to home caregivers, who must be reliable and well-informed [23]. Targeted interventions, including psychological support programs and remote monitoring technologies, are essential to reduce caregiver burden and improve sustainability of care.

Although most physicians reported referring patients for TCRT, nearly half indicated that fewer than one-quarter of referred patients ultimately received therapy—a finding consistent with national data showing significant attrition between referral and treatment [14]. Patient hesitancy or reluctance was the most frequently cited barrier physicians encountered when considering TCRT referral, suggesting that attrition begins before the referral itself, at the point of patient–physician discussion. In contrast, among patients who ultimately received TCRT, 88% reported no challenges in accessing therapy. This apparent discrepancy likely reflects survivorship bias: patients who initiated TCRT represent a selected group who overcame referral, eligibility, and logistical barriers, whereas those who did not proceed are not captured in the patient survey. Additionally, not all referred patients are ultimately eligible for TCRT, as clinical factors such as disease progression or organ dysfunction may account for a proportion of physician-reported attrition.

Community physicians may lack the specialized knowledge to adequately counsel patients about TCRT risks, benefits, and logistics, contributing to patient uncertainty. Virtual consultation between community physicians and TCRT specialists could help address this gap by enabling informed pre-referral discussions, building on established shared-care frameworks that have successfully facilitated transplant and cellular therapy referrals in underserved populations [24,25,26,27].

When asked about the role of location in TCRT access, 74% of physicians indicated that geography plays a moderate or major role. Accordingly, improved transportation and logistical support for patients was the most frequently selected recommendation for improving TCRT access in communities. Studies show that expanding access beyond academic hospitals to include community multispecialty hospitals and specialty oncology networks can reduce travel time by 23% and distance by 30% [28]. Beyond access challenges, nearly one-third of physicians reported discomfort managing patients post-TCRT, highlighting the need for targeted educational tools, including best-practice handbooks and webinars that community oncologists have specifically requested.

This study has several limitations. The cross-sectional design and single-institution setting preclude causal inference and may limit generalizability, though findings are relevant to similar comprehensive cancer centers delivering TCRT. The internally developed survey instruments, while reviewed for content validity, were not formally validated. Self-reported TCRT utilization was not verified through medical records, introducing potential recall and social desirability bias. Misclassification of treatment exposure may have occurred if patients did not accurately recall or recognize specific TCRT modalities received. Although the utilization analysis was restricted to patients reporting prior myeloma treatment, limited familiarity with TCRT terminology among some respondents suggests that reported utilization rates should be interpreted with caution. Additionally, “I don’t know” or missing responses regarding TCRT utilization were more frequent among Black patients and those with lower educational attainment, which may further limit the precision of utilization estimates in these subgroups. The underrepresentation of Black patients among respondents (18% vs. approximately 30% of our MM population) represents participation bias. Because patient surveys were distributed through MyChart, individuals with lower digital access or lower engagement with electronic health systems may have been less likely to participate, potentially contributing to selection bias in the study population. Survey non-respondents may have different treatment experiences, and our findings cannot address disparities among those who did not participate. The caregiver survey was limited to those whose care recipients had received or were receiving TCRT and did not distinguish between CAR T-cell and bispecific antibody recipients, therapies with differing long-term caregiver demands. The physician sample was small and recruited through a convenience sample of community physicians within our referral network, which may limit generalizability. Respondents may represent physicians with stronger engagement with tertiary referral centers, potentially introducing selection bias.

## 5. Conclusions

This multi-stakeholder survey identified interconnected barriers to TCRT access across patients, caregivers, and community physicians. Awareness gaps were more pronounced among Black patients and those with lower educational attainment, although TCRT utilization among previously treated patients was similar across racial groups at our center. Caregivers reported substantial emotional and time burdens, and physicians identified patient hesitancy as the primary barrier to successful referrals. These findings demonstrate that barriers to TCRT extend beyond treatment availability and encompass patient education, caregiver support, and coordination between community and specialized care settings. Improving equitable access will require accessible patient education, structured caregiver resources, community physician support, and enhanced referral and logistical pathways. Future multi-institutional studies should evaluate whether targeted interventions addressing these barriers can improve awareness, reduce disparities, and promote equitable TCRT access and should characterize caregiver burden specific to each TCRT modality.

## Figures and Tables

**Table 1:** Demographic Characteristics of Patient Respondents.

	Black N = 62	White and Others N = 284	Total N = 346
Age, years			
<60	2 (35%)	51 (18%)	73 (21%)
≥60	40 (65%)	232 (82%)	272 (79%)
Left Blank	0 (0%)	1 (0%)	1 (0%)
Sex assigned at birth			
Male	28 (45%)	165 (58%)	193 (56%)
Female	33 (53%)	116 (41%)	149 (43%)
Other/Not Answered	1 (2%)	3 (1%)	4 (1%)
Ethnicity			
Hispanic or Latino	0 (0%)	4 (1%)	4 (1%)
Not Hispanic or Latino	49 (79%)	250 (88%)	299 (86%)
Unknown/Not Answered	13 (21%)	30 (11%)	43 (12%)
Highest level of education completed			
High school or less	14 (23%)	45 (16%)	59 (17%)
More than high school	47 (76%)	235 (83%)	282 (82%)
Left blank	1 (2%)	4 (1%)	5 (1%)
Type of residence			
Urban/suburban area	44 (71%)	191 (67%)	235 (68%)
Rural area	10 (16%)	82 (29%)	92 (27%)
Unknown/Not Answered	8 (13%)	11 (4%)	19 (5%)

**Table 2:** Demographic Characteristics of Caregiver Respondents.

	Total N = 51
Age, years	
25–44	8 (16%)
45–64	11 (22%)
65+	32 (63%)
Sex assigned at birth	
Male	12 (24%)
Female	38 (75%)
Left Blank	1 (2%)
Race	
White or Caucasian	44 (86%)
Black or African American	6 (12%)
Other	1 (2%)
Ethnicity	
Hispanic or Latino	1 (2%)
Not Hispanic or Latino	47 (92%)
Prefer not to answer/Unknown	3 (6%)
Employment status	
Employed	16 (31%)
Caring for home or family	2 (4%)
Retired	32 (63%)
Left Blank	1 (2%)
Relationship with patient	
Spouse/partner	42 (82%)
Parent	1 (2%)
Sibling	3 (6%)
Child	5 (10%)
Same residence as patient	
Yes	45 (88%)
No	6 (12%)
Highest level of education completed	
High school or less	6 (12%)
More than high school	45 (88%)

## Data Availability

The deidentified survey data are available from the corresponding author upon reasonable request.

## Supplementary Materials

The following supporting information can be downloaded at: https://www.mdpi.com/article/10.3390/cancers18152412/s1, Table S1. Comparison of demographic characteristics between the target population and TCRT study respondents. Table S2. Concerns related to participation in clinical trials among Black (N = 62) and non-Black (N = 284) patient respondents. Multiple responses were permitted. Table S3. Individual logistic regression model results for the outcome of TCRT utilization in N = 254 patients who were defined to be eligible for TCRT. Race and education were the main covariates of interest, while age, sex, area and insurance were other available factors evaluated for association with reported TCRT utilization. Table S4. Patterns of missingness/“I don’t know” response for key outcomes in patients by race group. Table S5. Patterns of missingness/“I don’t know” response for key outcomes in patients by education group. Figure S1. Reported impact of caregiving during care recipients’ TCRT treatment. Figure S2. Recommendations for improving TCRT access in communities. Figure S3. Distributed multiple myeloma patient survey. Figure S4. Distributed caregiver survey. Figure S5. Distributed community physician survey.
