# Supplementary material for: Access to T-Cell Redirecting Therapies in Multiple Myeloma: Patient, Caregiver, and Physician Perspectives on Awareness and Barriers
Source: Cancers (Basel). 2026 Jul 27;18(15):2412. doi: 10.3390/cancers18152412 (PMC13465369; doi:10.3390/cancers18152412)
Supplement: Supplementary file 1 [file cancers-18-02412-s001.zip › cancers-4432131-supplementary.pdf]

**Supplemental Tables and Figures**

Table S1. Comparison of demographic characteristics between the target population and TCRT study respondents.

| | N=1770 | TCRT Study Numbers |
|---|---|---|
| Age | | |
| <60 | 347 (19.6%) | 73 (21%) |
| >=60 | 1423 (80.4%) | 272 (79%) |
| Gender | | |
| Male | 970 (54.8%) | 193 (56%) |
| Female | 800 (45.2%) | 149 (43%) |
| | N=1215 | |
| Race | | |
| Black | 413 (34.0%) | 62 (17.9%) |
| Non-Black | 802 (66.0%) | 284 (82.1%) |
| Ethnicity | | |
| Hispanic | 33 (2.7%) | 4 (1%) |
| Not Hispanic | 1164 (95.8%) | 299 (86%) |
| Not specified (or unknown/not answered) | 18 (1.5%) | 43 (12%) |

Table S2. Concerns related to participation in clinical trials among Black (N=62) and non-Black (N=284) patient respondents. Multiple responses were permitted.

| Do you have any concerns about participating in clinical trial(s)? | Black (N=62) | Non-Black (N=284) | Overall (N=346) |
|---|---|---|---|
| Yes | 19 (31%) | 66 (23%) | 85 (25%) |
| Difficulty understanding lengthy informed consent | 3 (5%) | 2 (1%) | 5 (1%) |
| Distrust in clinical trials or research | 3 (5%) | 9 (3%) | 12 (3%) |
| Other | 2 (3%) | 11 (4%) | 13 (4%) |
| Transportation concerns | 3 (5%) | 16 (6%) | 19 (5%) |
| Financial concerns (e.g. cost, insurance) | 10 (16%) | 20 (7%) | 30 (9%) |
| Lack of understanding about the treatment | 11 (18%) | 27 (10%) | 38 (11%) |
| Concerns about trial quality of life | 14 (23%) | 45 (16%) | 59 (17%) |
| Fear of side effects or complications | 15 (24%) | 46 (16%) | 61 (18%) |
| No concerns | 43 (69%) | 213 (75%) | 256 (74%) |
| Left blank | 0 (0%) | 5 (2%) | 5 (1%) |

Table S3. Individual logistic regression model results for the outcome of TCRT utilization in N=254 patients who were defined to be eligible for TCRT. Race and education were the main covariates of interest, while age, sex, area and insurance were other available factors evaluated for association with reported TCRT utilization.

Abbreviations: TCRT – T-cell redirecting therapies; OR – odds ratio; CI – confidence interval; HS – high school

| Individual Factor | OR (95% CI) | p-value |
|---|---|---|
| Race (Black vs non-Black) | 1.05 (0.55, 2.02) | 0.884 |
| Education (>HS vs HS or less) | 2.23 (1.00, 4.93) | 0.049 |
| Age group (≥60 vs <60 years) | 0.99 (0.53, 1.86) | 0.984 |
| Sex (Female vs Male) | 1.19 (0.71, 2.00) | 0.507 |
| Area (Rural vs Urban/Suburban) | 0.71 (0.39, 1.30) | 0.272 |
| Insurance (Commercial/Private vs Other) | 1.00 (0.58, 1.71) | 0.988 |

Table S4. Patterns of missingness / "I don't know" response for key outcomes in patients by race group.

Abbreviations: TCRT – T-cell redirecting therapies

| Total Patient Respondents | Black (N=62) | Non-Black (N=284) | Overall (N=346) |
|---|---|---|---|
| Left blank: Are you aware of CAR T-cell therapy or bispecific antibodies as treatment options for multiple myeloma? | 1 (2%) | 5 (2%) | 6 (2%) |
| Left blank: Do you have any concerns about participating in clinical trial(s)? | 0 (0%) | 5 (2%) | 5 (1%) |
| "Eligible" for TCRT Respondents | Black (N=46) | Non-Black (N=208) | Overall (N=254) |
| I don't know / Left blank: Have you received CAR T-cell therapy or bispecific antibodies as a treatment option? | 9 (20%) | 25 (12%) | 34 (13%) |
| Reported receiving TCRT | Black (N=18) | Non-Black (N=79) | Overall (N=97) |
| I don't know / Left blank: If you received CAR T-cell therapy or bispecific antibodies, was this treatment part of a clinical trial? | 3 (17%) | 5 (6%) | 8 (8%) |
| Left blank: Did you experience any challenges accessing CAR T-cell therapy or bispecific antibody therapy? | 0 (0%) | 2 (3%) | 2 (2%) |

| | | | |
|---|---|---|---|
| Left blank: Would you recommend CAR T-cell therapy or bispecific antibody therapy to others with multiple myeloma? | 1 (6%) | 1 (1%) | 2 (2%) |

Table S5. Patterns of missingness / "I don't know" response for key outcomes in patients by education group.

Abbreviations: TCRT – T-cell redirecting therapies; HS – high school

| Total Patient Respondents | HS or Less (N=59) | >HS (N=282) | Overall (N=341) |
|---|---|---|---|
| Left blank: Are you aware of CAR T-cell therapy or bispecific antibodies as treatment options for multiple myeloma? | 2 (3%) | 3 (1%) | 5 (1%) |
| Left blank: Do you have any concerns about participating in clinical trial(s)? | 2 (3%) | 3 (1%) | 5 (1%) |
| "Eligible" for TCRT Respondents | HS or Less (N=38) | >HS (N=213) | Overall (N=251) |
| I don't know / Left blank: Have you received CAR T-cell therapy or bispecific antibodies as a treatment option? | 10 (26%) | 23 (11%) | 33 (13%) |
| Reported receiving TCRT | HS or Less (N=9) | >HS (N=87) | Overall (N=96) |
| I don't know / Left blank: If you received CAR T-cell therapy or bispecific antibodies, was this treatment part of a clinical trial? | 2 (22%) | 6 (7%) | 8 (8%) |
| Left blank: Did you experience any challenges accessing CAR T-cell therapy or bispecific antibody therapy? | 0 (0%) | 4 (5%) | 4 (4%) |
| Left blank: Would you recommend CAR T-cell therapy or bispecific antibody therapy to others with multiple myeloma? | 0 (0%) | 2 (2%) | 2 (2%) |

Figure S1. Reported impact of caregiving during care recipients' TCRT treatment

Abbreviations: TCRT – T-cell redirecting therapies

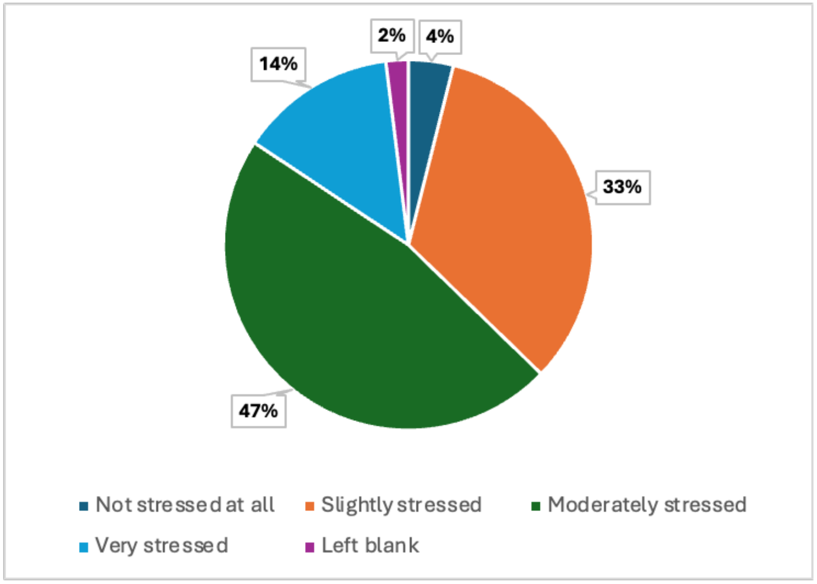


Figure S2. Recommendations for improving TCRT access in communities

Abbreviations: TCRT – T-cell redirecting therapies

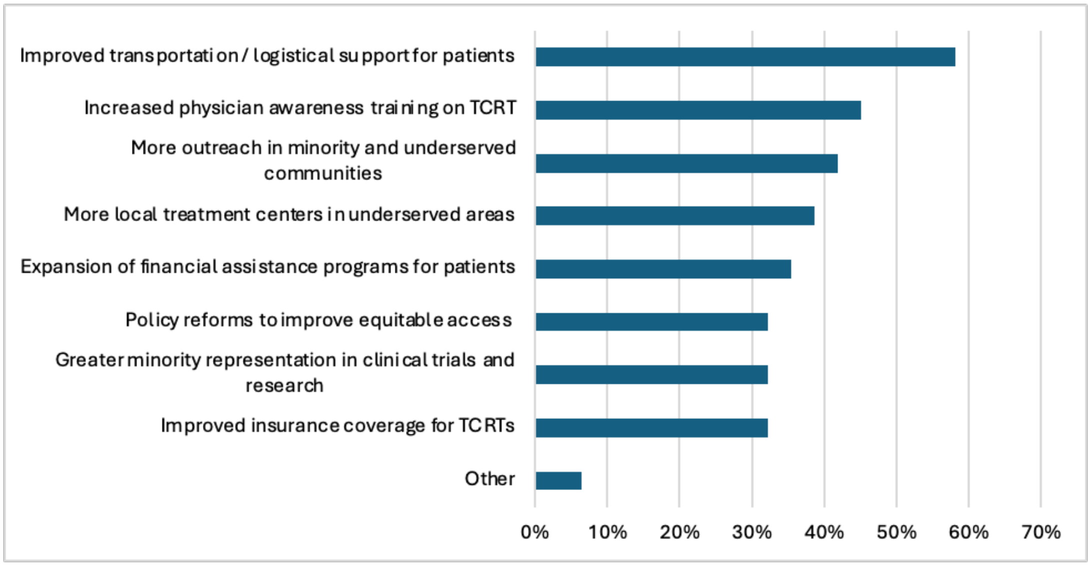

Figure S3. Distributed multiple myeloma patient survey



# Multiple Myeloma Patient Perspective

This survey is intended to be completed by patients who have a diagnosis of multiple myeloma.

## Section 1: Patient Demographics and Background

| | | |
|---|---|---|
| 1 | Do you have a diagnosis of multiple myeloma? | ◯ Yes<br>◯ No |
| 2 | What is your age? | ◯ 18-29<br>◯ 30-39<br>◯ 40-49<br>◯ 50-59<br>◯ 60-69<br>◯ 70-79<br>◯ 80 years or older |
| 3 | What is your sex assigned at birth? | ◯ Male<br>◯ Female<br>◯ Other<br>◯ Prefer not to answer |
| 4 | What is your race? | ◯ White or Caucasian<br>◯ Black or African American<br>◯ Asian<br>◯ Native Hawaiian or Other Pacific Islander<br>◯ American Indian or Alaska Native<br>◯ Other<br>◯ Prefer not to answer |
| | Specify if other | __________________________ |
| 5 | What is your ethnicity? | ◯ Hispanic or Latino<br>◯ Not Hispanic or Latino<br>◯ Unknown<br>◯ Prefer not to answer |
| 6 | How long have you been diagnosed with multiple myeloma? | ◯ Less than 1 year<br>◯ 1-3 years<br>◯ 4-6 years<br>◯ 7-10 years<br>◯ More than 10 years |
| 7 | What is the highest level of education you have completed? | ◯ Less than high school<br>◯ Some high school<br>◯ High school diploma or equivalent<br>◯ Some college or associate degree<br>◯ Bachelor's degree<br>◯ Graduate or professional degree |
| 8 | What is your annual household income? | ◯ Less than $25,000<br>◯ $25,000-$49,999<br>◯ $50,000-$74,999<br>◯ $75,000-$99,999<br>◯ $100,000 or more<br>◯ Prefer not to answer |

| | | |
|---|---|---|
| 9 | In which region do you currently reside? | ◯ Northeast<br>◯ Midwest<br>◯ South<br>◯ West<br>◯ I do not reside in the United States<br>◯ Prefer not to answer |
| 10 | What type of residence do you live in? | ◯ Urban area<br>◯ Suburban area<br>◯ Rural area<br>◯ Prefer not to answer |
| 11 | What is your primary type of insurance? | ◯ Uninsured/self-pay<br>◯ Medicaid<br>◯ Medicare<br>◯ VA<br>◯ Commercial / private insurance<br>◯ Prefer not to answer |

## Section 2: Treatment History and Awareness

| | | |
|---|---|---|
| 12 | Have you received or are you receiving treatment(s) for your multiple myeloma? | ◯ Yes<br>◯ No |
| 13 | Are you currently receiving your first treatment for multiple myeloma? | ◯ Yes<br>◯ No |
| 14 | Are you currently receiving treatment for relapsed multiple myeloma (myeloma was previously treated and has come back)? | ◯ Yes<br>◯ No |
| 15 | Are you aware of CAR T-cell therapy or bispecific antibodies as treatment options for multiple myeloma?<br><br>Examples of commercial CAR T-cell therapy: Carvykti, Abecma, ide-cel, cilta-cel.<br><br>Examples of investigational CAR T-cell therapy: anito-cel, arlo-cel<br><br>Examples of commercial bispecific antibody treatments: teclistamab, talquetamab, elranatamab, Tecvayli, Talvey, Elrexfio.<br><br>Examples of investigational bispecific antibody treatments: etentamig, ABBV-383, linvoseltamab | ◯ Yes, I am aware of both<br>◯ Yes, I am aware of CAR T-cell therapy<br>◯ Yes, I am aware of bispecific antibodies<br>◯ No, I am not aware of either |
| 16 | Do you have any concerns about participating in clinical trial(s)? | ◯ Yes<br>◯ No |
| | If yes, please check all concerns that apply | ☐ Fear of side effects or complications<br>☐ Distrust in clinical trials or research<br>☐ Lack of understanding about the treatment<br>☐ Financial concerns (e.g., cost, insurance)<br>☐ Transportation concerns<br>☐ Concerns about how the trial might affect my quality of life<br>☐ Difficulty understanding language/details of lengthy informed consent<br>☐ Other |
| | If other concerns about clinical trial participation, please specify | __________________________________ |

**Section 3: Experience with CAR T-cell Therapy and Bispecific Antibodies**

17 Have you received CAR T-cell therapy or bispecific antibodies as a treatment option?

Examples of commercial CAR T-cell therapy: Carvykti, Abecma, ide-cel, cilta-cel.

Examples of investigational CAR T-cell therapy: anito-cel, arlo-cel

Examples of commercial bispecific antibody treatments: teclistamab, talquetamab, elranatamab, Tecvayli, Talvey, Elrexfio.

Examples of investigational bispecific antibody treatments: etentamig, ABBV-383, linvoseltamab

- ◯ Yes, I have received CAR T-cell therapy
- ◯ Yes, I have received bispecific antibodies
- ◯ Yes, I have received both
- ◯ No, I have not received either
- ◯ I don't know

18 Are you currently being considered for CAR T-cell therapy or bispecific antibodies as a treatment option?

- ◯ Yes
- ◯ No
- ◯ I don't know

## Section 3.1 For Patients Who Have Received CAR T-cell Therapy or Bispecific Antibodies

19 If you received CAR T-cell therapy or bispecific antibodies, was this treatment part of a clinical trial?

- ◯ Yes, it was part of a clinical trial
- ◯ No, it was not part of a clinical trial
- ◯ I don't know

20 How long ago did you receive CAR T-cell therapy or bispecific antibody therapy?

- ◯ Less than 3 months ago
- ◯ 3-6 months ago
- ◯ 7-12 months ago
- ◯ More than a year ago

21 Did you experience any challenges accessing CAR T-cell therapy or bispecific antibody therapy?

- ◯ Yes
- ◯ No

Please check all challenges that apply

- ☐ Difficulty finding an authorized treatment center
- ☐ Travel/logistical challenges
- ☐ Need to relocate closer to an authorized treatment center
- ☐ Delays in treatment initiation or waiting for results
- ☐ Insurance or financial challenges
- ☐ Lack of support from healthcare team
- ☐ Difficulty securing a caregiver
- ☐ Other

If other, please specify the challenges you faced?

__________________________________

22 Would you recommend CAR T-cell therapy or bispecific antibody therapy to others with multiple myeloma?

- ◯ Yes, I would recommend it
- ◯ Yes, with some reservations
- ◯ No, I would not recommend it
- ◯ Not sure

**Section 3.2 For Patients Who Have NOT Received CAR T-cell Therapy or Bispecific Antibodies**

| | | |
|---|---|---|
| 23 | Have you previously been offered CAR T cell therapy or bispecific antibody treatment? | ◯ Yes<br>◯ No |
| | If yes, check all reasons for why you did not receive treatment when initially offered | ☐ Concerns about potential side effects and unfavorable outcome<br>☐ Lack of information or understanding<br>☐ Personal or family preferences<br>☐ Financial considerations<br>☐ Caregiver challenges<br>☐ Transportation challenges<br>☐ Previous treatment experience<br>☐ Burden of relocation closer to an authorized treatment center<br>☐ Not ready to commit<br>☐ I was not a good candidate for the treatment according to my physician<br>☐ Other |
| | If other, please specify | ______________________________ |

## Section 3.3 For Patients Who Are Currently Being Considered for CAR T-cell Therapy or Bispecific Antibodies (but haven't previously received)

| | | |
|---|---|---|
| 24 | Would you consider receiving CAR T-cell therapy or bispecific antibody treatment if your doctor recommended it? | ◯ Yes<br>◯ No<br>◯ Maybe, depending on more information |
| 25 | What factors would influence your decision in receiving CAR T-cell therapy or bispecific antibody treatment? (Select all that apply) | ☐ Physician recommendation<br>☐ Side effects<br>☐ Effectiveness of the treatment<br>☐ Cost and insurance coverage<br>☐ Access to treatment<br>☐ Knowledge about the therapy<br>☐ Need for inpatient monitoring<br>☐ Other |
| | If other, please specify | __________________________ |

Figure S4. Distributed caregiver survey



# Caregiver Perspective

This survey is intended to be completed by caregivers of multiple myeloma patients who have received or are currently receiving CAR-T cell therapy or bispecific antibody treatment.

## Section 1: Caregiver Demographics and Background

1 Are you a caregiver for a patient with multiple myeloma who has received or is currently receiving CAR-T cell therapy or Bispecific Antibody treatment?

Examples of commercial CAR T-cell therapy: Carvykti, Abecma, ide-cel, cilta-cel.

Examples of investigational CAR T-cell therapy: anito-cel, arlo-cel

Examples of commercial bispecific antibody treatments: teclistamab, talquetamab, elranatamab, Tecvayli, Talvey, Elrexfio.

Examples of investigational bispecific antibody treatments: etentamig, ABBV-383, linvoseltamab

- ◯ Yes
- ◯ No

2 What is your relationship to the patient? (Select one)

- ◯ Spouse/Partner
- ◯ Parent
- ◯ Sibling
- ◯ Child
- ◯ Friend
- ◯ Neighbor
- ◯ Coworker
- ◯ Other

If other, please specify

__________________________________

3 How old are you?

- ◯ 18-24
- ◯ 25-34
- ◯ 35-44
- ◯ 45-54
- ◯ 55-64
- ◯ 65 or older

4 What is your sex assigned at birth?

- ◯ Male
- ◯ Female
- ◯ Other
- ◯ Prefer not to answer

5 What is your race?

- ◯ White or Caucasian
- ◯ Black or African American
- ◯ Asian
- ◯ Native Hawaiian or Other Pacific Islander
- ◯ American Indian or Alaska Native
- ◯ Other
- ◯ Prefer not to answer

If other, please specify

__________________________________

| | | |
|---|---|---|
| 6 | What is your ethnicity? | ◯ Hispanic or Latino<br>◯ Not Hispanic or Latino<br>◯ Unknown<br>◯ Prefer not to answer |
| 7 | What is your employment status? | ◯ Employed<br>◯ Caring for home of family<br>◯ Unable to work due to illness or disability<br>◯ Retired<br>◯ Student<br>◯ Other |
| 8 | Are you living in the same residence at the patient? | ◯ Yes<br>◯ No |
| 9 | What is the highest level of education you have completed? | ◯ Less than high school<br>◯ Some high school<br>◯ High school diploma or equivalent<br>◯ Some college<br>◯ Associate degree or vocational school<br>◯ College graduate/Bachelor's degree<br>◯ Graduate or professional degree |
| 10 | On average, how many hours per week do you spend providing care for the patient during the first month of CAR-T cell therapy or Bispecific Antibody treatment? | ◯ Less than 5 hours per week<br>◯ 5-10 hours per week<br>◯ 11-20 hours per week<br>◯ 21-30 hours per week<br>◯ 31-40 hours per week<br>◯ More than 40 hours per week |

## Section 2: Caregiver Experience with CAR-T cell therapy or Bispecific Antibody Treatment

11 What was your level of knowledge about CAR-T or bispecific antibody therapies before the patient started treatment?
- ◯ Very knowledgeable
- ◯ Somewhat knowledgeable
- ◯ Heard of it, but not familiar
- ◯ No knowledge

12 How would you rate the amount of information provided to you by the healthcare team about the patient's CAR-T cell therapy or Bispecific antibody treatment before it started?
- ◯ Very sufficient
- ◯ Somewhat sufficient
- ◯ Somewhat insufficient
- ◯ Not at all sufficient

13 How well prepared did/do you feel to support the patient during the treatment process?
- ◯ Very well prepared
- ◯ Somewhat prepared
- ◯ Not well prepared
- ◯ Not at all prepared

14 Did you experience any challenges finding a treatment center for CAR-T cell therapy or Bispecific Antibody treatment?
- ◯ Yes
- ◯ No

15 Were you taught how to monitor for CRS and ICANs as side effects from CAR T cell therapy or bispecific antibody treatments?
- ◯ Yes
- ◯ No

16 Have you encountered any significant side effects in the patient that have been difficult to manage?
- ◯ Yes
- ◯ No

If yes, check all that apply
- ☐ Cytokine release syndrome (CRS) - fever, chills, difficulty breathing, body aches, low blood pressure, dizziness, low oxygen level, difficulty breathing
- ☐ Neurotoxicity - change in behavior, difficulty carrying a coherent conversation, change in personality, headache, tremors, trouble keeping attention, showing confusion
- ☐ Fatigue
- ☐ Pain
- ☐ Low blood counts / need for transfusions
- ☐ Repeated infections
- ☐ Weight loss
- ☐ Loss of appetite
- ☐ GI problems (nausea, vomiting, diarrhea)
- ☐ Change in taste
- ☐ Skin changes
- ☐ Other

If other side effect, please specify

__________________________________

**Section 3: Impact of Treatment on Caregiver**

17 Which of the following have been affected due to your role as a caregiver during the patient's CAR T-cell therapy or bispecific antibody treatment? (Select all that apply)

- ☐ Mental or emotional wellness
- ☐ Financial wellness
- ☐ Social wellness
- ☐ Physical wellness
- ☐ Work performance
- ☐ None

18 How would you describe the emotional impact of caregiving during the patient's CAR T-cell therapy or bispecific antibody treatment?

- ◯ Not stressed at all
- ◯ Slightly stressed
- ◯ Moderately stressed
- ◯ Very stressed

19 If you are/were employed, have you experienced any work-related challenges during your role as caregiver during the patient's treatment with CAR-T cell therapy or Bispecific Antibodies?

- ◯ Went in late, left early or took time off
- ◯ Went from full-time to part-time work
- ◯ Received a warning about attendance or performance
- ◯ Turned down a promotion
- ◯ Quit working entirely
- ◯ Retired early
- ◯ Not applicable

20 What have been the challenging aspects of caregiving during CAR T cell therapy or bispecific antibody treatment? (Select all that apply)

- ☐ No major challenges
- ☐ Managing side effects (e.g., CRS, neurotoxicity, infections)
- ☐ Coordinating and attending medical appointments
- ☐ Understanding what to do in an emergency and who to contact
- ☐ Transportation and driving to treatment centers
- ☐ Burden of relocation closer to authorized treatment center
- ☐ Lack of support from healthcare providers
- ☐ Financial burden (e.g., out-of-pocket costs, insurance issues)
- ☐ Emotional distress (e.g., seeing the patient suffer)
- ☐ Not enough time for personal care/needs
- ☐ Isolation from family, friends or community
- ☐ Lack of caregiver resources or training
- ☐ Work absences / work disruptions / time away from work
- ☐ Other

If other challenges, please specify

__________________________________

21 How supported do you feel by the healthcare team during the patient's CAR-T or Bispecific Antibody treatment?

- ◯ Very supported
- ◯ Somewhat supported
- ◯ Not supported

22 Have you received any support or counseling as a caregiver during this treatment process?

- ◯ Yes
- ◯ No

22 If yes, check all that apply

- ☐ From support groups or community resources
- ☐ From the healthcare team
- ☐ From my employer
- ☐ From online forums
- ☐ From the pharmaceutical company (for example for lodging and food)

Figure S5. Distributed community physician survey



# Community Physician Perspective

This survey is intended to be completed by community physicians who treat multiple myeloma patients and whose primary practice cannot provide CAR T cell therapy or step up dosing of bispecific antibody treatments.

## Section 1: Physician Demographics & Practice Information

1 Do you see and/or treat patients with multiple myeloma (even if infrequently)?

○ Yes
○ No

2 Are you able to provide CAR T-cell therapy or step-up dosing of bispecific antibody treatments for multiple myeloma at your primary practice?

Examples of commercial CAR T-cell therapy: Carvykti, Abecma, ide-cel, cilta-cel.

Examples of investigational CAR T-cell therapy: anito-cel, arlo-cel

Examples of commercial bispecific antibody treatments: teclistamab, talquetamab, elranatamab, Tecvayli, Talvey, Elrexfio.

Examples of investigational bispecific antibody treatments: etentamig, ABBV-383, linvoseltamab

○ Yes
○ No

3 What is your medical specialty?

☐ Hematology
☐ Medical Oncology
☐ Internal Medicine
☐ Other

If other, please specify your specialty

__________________________________

4 How many years have you been practicing medicine?

○ 0-5 years
○ 6-10 years
○ 11-20 years
○ 21+ years

5 What percentage of your multiple myeloma patients are from marginalized communities (e.g., African American, Hispanic/Latino, Indigenous, low-income)?

○ Less than 10%
○ 10-25%
○ 26-50%
○ More than 50%

## Section 2: Awareness and Utilization of CAR-T Cell Therapy or Bispecific Antibody treatment

6 How familiar are you with CAR-T cell therapy or Bispecific Antibody (BsAb) treatment for multiple myeloma?

- ◯ Very familiar - I actively refer and discuss it with patients
- ◯ Somewhat familiar - I know the basics but do not frequently refer patients
- ◯ Not familiar - I have little to no knowledge about CAR-T cell therapy or BsAb

7 How often do you discuss CAR-T cell therapy or Bispecific Antibody (BsAb) as a treatment option with your patients with multiple myeloma?

- ◯ Always, if they are eligible
- ◯ Sometimes, depending on the patient's condition
- ◯ Rarely
- ◯ Never

8 Have you referred patients with multiple myeloma to a specialized center for CAR-T cell therapy or Bispecific Antibody (BsAb) treatment?

- ◯ Yes, multiple times (≥5)
- ◯ Yes, but only a few times (< 5)
- ◯ No

9 Of those referred, what proportion of the patients actually received CAR-T cell therapy or Bispecific Antibody (BsAb) treatment?

- ◯ 76-100%
- ◯ 51-75%
- ◯ 26-50%
- ◯ 25% or less

10 What patient-related factors do you consider when deciding whether to recommend CAR-T cell therapy or Bispecific Antibody treatment?
(Check all that apply)

- ☐ Age
- ☐ Presence of comorbidities
- ☐ Patient preference or willingness to undergo treatment
- ☐ Performance status (e.g., physical functioning)
- ☐ Disease prognosis
- ☐ Prior treatment history and response

11 Have you taken care of patients after CAR-T therapy or after step-up dosing of Bispecific Antibodies (BsAb)?

- ◯ Yes, post-CAR-T therapy
- ◯ Yes, after step-up dosing of Bispecific Antibodies
- ◯ I have cared for patients after both CAR-T therapy and step-up dosing of Bispecific Antibodies
- ◯ No, I have not cared for patients post-CAR-T or post-BsAb step up dosing

12 How comfortable are you caring for patients treated with CAR-T cell therapy or Bispecific Antibodies (BsAb)?

- ◯ Very comfortable - I am confident in managing these patients and their treatment plans
- ◯ Somewhat comfortable - I can manage basic care but may need additional support for complications
- ◯ Not very comfortable - I have limited experience and need more training or resources
- ◯ Not comfortable at all - I am not familiar with CAR-T cell therapy or BsAb therapy

13 What are some of the challenges you face when taking care of patients post-CAR-T or after step-up dosing of Bispecific Antibodies (BsAb)?
(Check all that apply)

- ☐ No significant challenges
- ☐ Managing complications such as cytokine release syndrome (CRS) or neurotoxicity
- ☐ Monitoring and treating infections due to immunosuppression
- ☐ Difficulty coordinating care with specialized treatment centers
- ☐ Addressing financial or insurance coverage issues for ongoing care
- ☐ Educating patients and caregivers about the therapy and potential side effects
- ☐ Ensuring proper follow-up care and patient adherence
- ☐ Managing long-term or delayed side effects
- ☐ Lack of in-house expertise and resources to handle complex side effects or complications
- ☐ Limited access to specialists for guidance on complex cases
- ☐ Lack of clear instructions to me (primary oncologist) at discharge regarding follow-up

## Section 3: Barriers to Referring Patients for CAR-T Cell Therapy or Bispecific Antibody Treatment

14 What are the most significant barriers you encounter when considering a referral for CAR-T cell therapy or Bispecific Antibody treatment? (Check up to 3)

- ☐ Lack of familiarity with the complexity of these therapies
- ☐ Struggling to understand when to recommend these products
- ☐ Difficulty identifying a certified CAR T center
- ☐ Difficulty assisting with financial or insurance coverage issues
- ☐ High rate of denials by payers
- ☐ Patient hesitancy or reluctance to pursue treatment
- ☐ No time to explore eligibility criteria for CAR T cell therapy or BsAb
- ☐ Lengthy referral process and delays that impact patient outcomes
- ☐ Patient deterioration during the referral process prior to receiving CAR T cell therapy or BsAb
- ☐ Barriers to clinical trial access and enrollment
- ☐ Other

If other, please specify

__________________________________

15 How often do financial concerns prevent your patients from pursuing CAR-T cell therapy or Bispecific Antibody treatment?

- ○ Very often
- ○ Sometimes
- ○ Rarely
- ○ Never

16 Do you believe that racial disparities affect access to CAR-T cell therapy or Bispecific Antibody treatment?

- ○ Yes, significantly
- ○ Yes, somewhat
- ○ No
- ○ Unsure

17 Do you believe that socioeconomic disparities affect access to CAR-T cell therapy or Bispecific Antibody treatment?

- ○ Yes, significantly
- ○ Yes, somewhat
- ○ No
- ○ Unsure

18 What role does location play in access to CAR-T cell therapy or Bispecific Antibody treatment for patients?

- ○ A major role - many patients do not live near a treatment center
- ○ A moderate role - some patients struggle with transportation, but it's not a primary barrier
- ○ A minor role - most patients can find a way to access treatment if eligible
- ○ No role - location is not a factor in access

## Section 4: Improving Access and Equity

19 What do you believe would help improve CAR-T cell therapy and Bispecific Antibody (BsAb) access in communities?
(Check all that apply)

- ☐ More education and outreach in minority and underserved communities
- ☐ Increased physician awareness and training on CAR-T cell therapy and referral pathways
- ☐ Expansion of financial assistance programs for patients
- ☐ More local treatment centers in underserved areas
- ☐ Improved insurance coverage for CAR-T cell therapy and BsAb treatment
- ☐ Greater minority representation in clinical trials and research
- ☐ Improved transportation or logistical support for patients
- ☐ Policy reforms to improve equitable access to advanced therapies
- ☐ Other

If other, please specify

______________________________

20 Do you believe clinical trial enrollment requirements exclude many minority patients from CAR-T cell therapy or Bispecific Antibody (BsAb) access?

- ◯ Yes, significantly
- ◯ Yes, somewhat
- ◯ No, clinical trial criteria are fair/not prohibitive
- ◯ Unsure

21 Would you be interested in additional training or resources to help guide patients toward CAR-T cell therapy or Bispecific Antibody (BsAb)?

- ◯ Yes
- ◯ No
- ◯ Maybe, if it were easily accessible
